# CD38 in Adenosinergic Pathways and Metabolic Re-programming in Human Multiple Myeloma Cells: In-tandem Insights From Basic Science to Therapy

**DOI:** 10.3389/fimmu.2019.00760

**Published:** 2019-04-24

**Authors:** Alberto L. Horenstein, Cristiano Bracci, Fabio Morandi, Fabio Malavasi

**Affiliations:** ^1^Laboratory of Immunogenetics, Department of Medical Sciences, Turin, Italy; ^2^CeRMS, University of Torino, Turin, Italy; ^3^Stem Cell Laboratory and Cell Therapy Center, Istituto Giannina Gaslini, Genova, Italy

**Keywords:** CD38, multiple myeloma, metabolic reprogramming, adenosine, immunotherapy

## Abstract

Tumor microenvironments are rich in extracellular nucleotides that can be metabolized by ectoenzymes to produce adenosine, a nucleoside involved in controlling immune responses. Multiple myeloma, a plasma cell malignancy developed within a bone marrow niche, exploits adenosinergic pathways to customize the immune homeostasis of the tumor. CD38, a multifunctional protein that acts as both receptor and ectoenzyme, is overexpressed at all stages of myeloma. At neutral and acidic pH, CD38 catalyzes the extracellular conversion of NAD^+^ to regulators of calcium signaling. The initial disassembly of NAD^+^ is also followed by adenosinergic activity, if CD38 is operating in the presence of CD203a and CD73 nucleotidases. cAMP extruded from tumor cells provides another substrate for metabolizing nucleotidases to signaling adenosine. These pathways flank or bypass the canonical adenosinergic pathway subjected to the conversion of ATP by CD39. All of the adenosinergic networks can be hijacked by the tumor, thus controlling the homeostatic reprogramming of the myeloma in the bone marrow. In this context, adenosine assumes the role of a local hormone: cell metabolism is adjusted via low- or high-affinity purinergic receptors expressed by immune and bone cells as well as by tumor cells. The result is immunosuppression, which contributes to the failure of immune surveillance in cancer. A similar metabolic strategy silences immune effectors during the progression of indolent gammopathies to symptomatic overt multiple myeloma disease. Plasma from myeloma aspirates contains elevated levels of adenosine resulting from interactions between myeloma and other cells lining the niche and adenosine concentrations are known to increase as the disease progresses. This is statistically reflected in the International Staging System for multiple myeloma. Along with the ability to deplete CD38^+^ malignant plasma cell populations which has led to their widespread therapeutic use, anti-CD38 antibodies are involved in the polarization and release of microvesicles characterized by the expression of multiple adenosine-producing molecules. These adenosinergic pathways provide new immune checkpoints for improving immunotherapy protocols by helping to restore the depressed immune response.

## Multiple Myeloma

Multiple Myeloma (MM) is the second most common malignancy in hematology (1). MM is seem as the eventual outcome of selective pressures on different cell clones of malignant plasma cells (mPCs) (2) that grow in a hypoxic niche in the bone marrow (BM) (3). When oxygen consumption exceeds its supply from the vascular system, the hypoxic tumor environment favors molecular pathways that fuel tumor aggressiveness (4, 5). Cross talk among the distinct cellular components of the closed BM niche generates extracellular adenosine (ADO), thereby promoting tumor cell survival (6, 7). This occurs through the binding of ADO to purinergic receptors, which leads to the formation of complexes that function as autocrine/paracrine signals with immune regulatory activities.

Adenosine triphosphate (ATP), nicotinamide adenine dinucleotide (NAD^+^), and cyclic adenosine monophosphate (cAMP) are, the main intracellular purine molecules serving as leading adenosinergic substrates in the extracellular tumor microenvironment (TME) for generating ADO (8–10). Adenosinergic conversion, which can vary significantly according to the metabolic environment, is exploited by mPCs for migrating and homing to a protected niche and for evading the immune response (1). The expression of multiple specific P1 ADO receptors (ADORs) (11) in the niche completes the profile of a complex regulatory network, whose signals are translated into (i) down-regulation of the functions of most immune effector cells and (ii) enhancement of the activity of cells that suppress anti-tumor immune responses. Both effects facilitate the escape of mPCs from immune surveillance. A translational view of these findings suggests that finely-tuned ADO concentrations in the BM myeloma niche (12) contribute to symptomatic MM among patients with asymptomatic monoclonal gammopathy of undetermined significance (MGUS) and with smoldering multiple myeloma (SMM) (13, 14). Therefore, nucleotide-metabolizing ectoenzymes expressed by BM-resident cells and ADO production may acquire theragnostic relevance in the clinical outcome of MM. The present paper also reviews the contribution of metabolic reprogramming to the development of novel therapy options for MM.

## Adenosine Production within the BM Niche

### Purinome and Metabolic Reprogramming

**Figure 1:**
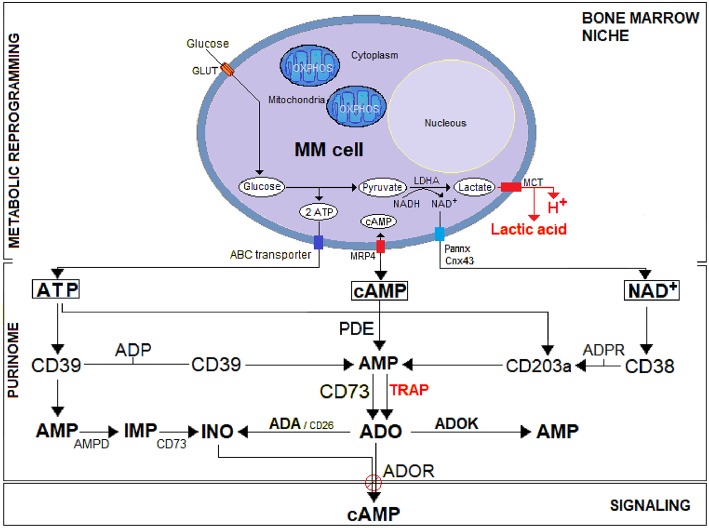
A schematic representation of the purinome in the MM environment under bone marrow niche metabolic reprogramming. Unlike normal cells, tumor cells (i.e., MM) utilize glycolysis instead of OXPHOS for metabolic reprogramming: most of the resulting pyruvate is catalyzed by lactate dehydrogenase A (LDH-A) to lactic acid simultaneously producing protons (H^+^). The efflux of lactic acid and H^+^ induces lactic acidosis generating an acidic TME (pH < 6.5). Cytoplasmic ATP, cAMP, and NAD^+^ actively secreted across nucleotide transporters (e.g., ABC transporter, Pannexin/Connexin channels, MRP4) or passively after cell lysis, are metabolized in the hypoxic acidic BM niche to ADO. The purinome (e.g., complex network of nucleotidic substrates, ectonucleotidases, signaling by-products, and purinergic receptors) operating in the BM niche exploit the classical pathway (CD39/CD73) for ATP substrate and the (CD38/CD203a/CD73) pathway for NAD^+^, flanked by an alternative (PDE/CD73) pathway that converts cAMP to AMP for generating the immunosuppressive ADO. Generated ADO binds to P1 purinergic ADO receptors (ADOR) and activates adenylyl cyclase, which catalyzes the formation of the intracellular second messenger cAMP. Eventually, ADO can also be inactivated at the cell surface by an ADA/CD26 complex that converts it into inosine (INO) or internalized by nucleoside transporters. The extracellular ATP breakdown follows under physiological conditions the classical ATP/ADP/AMP/ADO adenosinergic pathway. However, the presence of high ATP concentration in the TME lead AMP to be converted by AMP deaminase (AMPD) into inosine monophosphate (IMP), which in turn is dephosphorylated by 5′-NT/CD73 into INO.

A unique feature shared by the MGUS/SMM/MM stages of myeloma is the dependence of mPCs on BM microenvironmental signals. The BM niche provides a hypoxic habitat for interactions between mPCs and non-tumor immune and non-immune resident cells, yielding functional operating elements, or so-called purinome (e.g., nucleotide channels and transporters, nucleotides byproducts, nucleotide catabolizing ectoenzymes, molecular networks of nucleotide, and nucleoside receptor proteins) (15). Their physical connection is mediated by the plasmatic fluid, which links the different cell components of the purinome (Figure 1).

The MM grows in the BM, where mPCs are sheltered in a physically constrained niche containing osteoblasts (OBs), osteoclasts (OCs), stromal cells (SCs), and immune cells (e.g., T and B lymphocytes, NK, MDSC, among others). For their progressive expansion, mPCs overcome the hypoxic niche through a process of metabolic reprogramming based on hijacking the molecular mechanisms of normal cells to create an exclusive immunosuppressive frame. Metabolic adaptation of the cellular component of the BM niche induces a HIF1α-dependent glycolytic program, which increases CD73 and ADOR expression (16). Further, mPCs exploit local metabolic dysregulation, namely a shift from oxidative phosphorylation (OXPHOS) toward a glycolytic metabolism, to demand (i) supplementary sources of energy for a rapid growth; (ii) byproducts (e.g., ribose, glycerol and citrate, and non-essential amino acids) needed for biosynthetic pathways and (iii) an increase in enzymatic activities (e.g., LDH-A to regenerate NAD^+^) (17, 18). As shown in Figure 1, after consuming glucose at a higher rate than normal cells, MM cells secreted most of the derived byproducts as lactic acid, a phenomenon known as the “Warburg Effect” (19). Simultaneously, the generation of lactic acid and protons (H^+^) results in acid accumulation within MM cells. The intracellular metabolic adjustment is neutralized by the overexpression of a monocarboxylate transporter (MCT), resulting in a H^+^-linked co-transport of lactic acid across the plasma membrane, with increased extracellular acidity, known as lactic acidosis (Figure 1).

### Adenosine Production by Canonical and Alternative Ectoenzymatic Pathways

**Figure 2:**
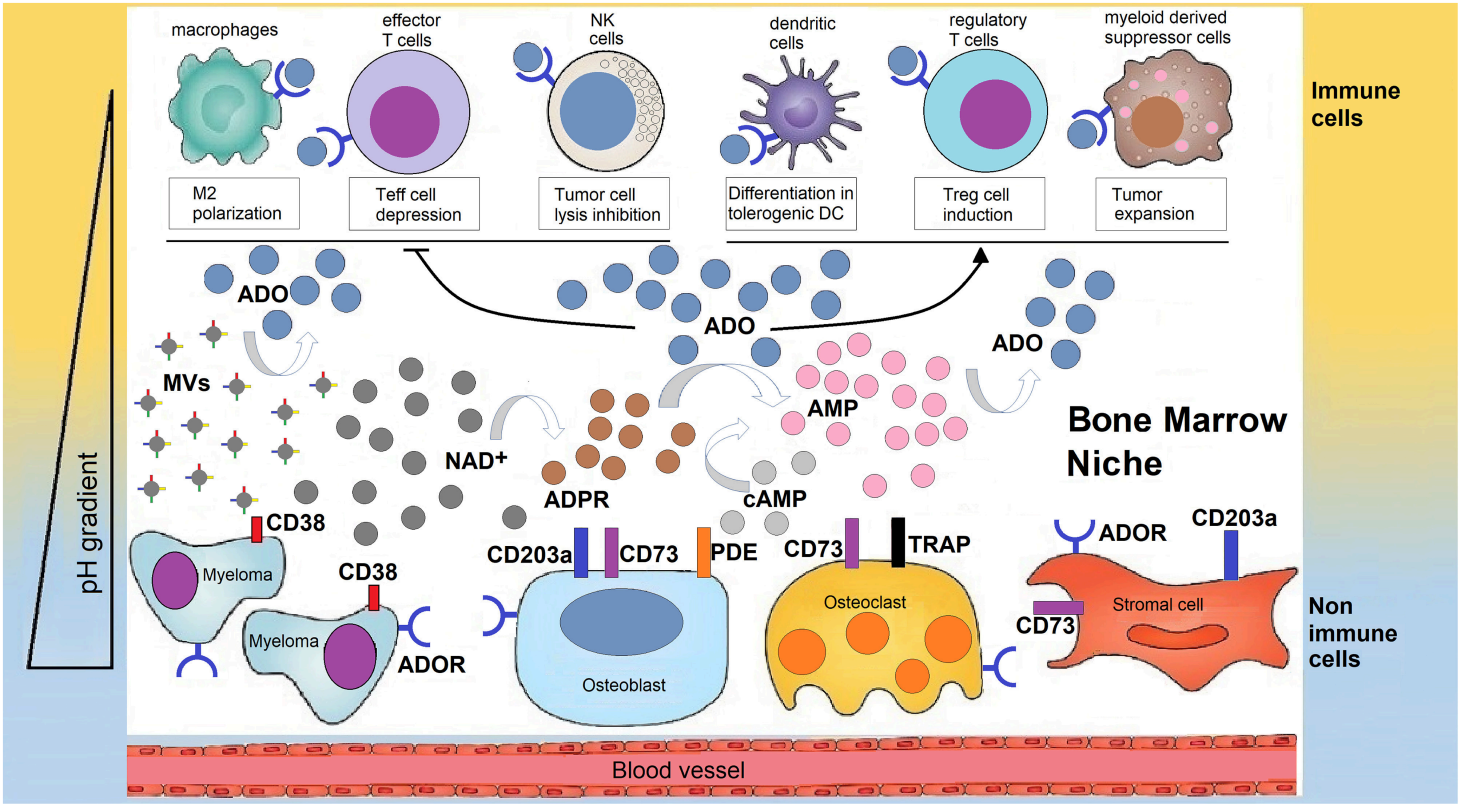
Human bone marrow sheltering malignant plasma cells, bone cells, and immune cell, supports the production of adenosine for the generation of a tolerant niche. In a closed BM niche, ADO is mainly obtained from NAD^+^ (and possibly from cAMP) which undergoes reaction through a multicellular chain of ectonucleotidases (CD38, CD203a, and CD73 or TRAP depending on pH status regulated by metabolic reprogramming). According to this view, NAD^+^ is disassembled into byproducts that flow in the BM plasma fluid within the myeloma niche, accumulating variable amounts of ADO. Most of ADO is taken-up by purinergic cell receptors (ADOR) expressed by bone cells or immune cells inside the niche. The outcome is either a block of the effectiveness of immune cells (Teff, NK, TAMs) that are capable of destroying tumor cells or that increase the number of regulatory T-cells (Tregs), mesenchymal derived stromal cells (MDSC), or dendritic cells (DC) which suppress immune cells from responding to the tumor.

The purinome exploits the metabolically reprogrammed niche to generate extracellular ADO which is locally produced by the multicellular network (Figure 2). ADO leads to tumor growth and skews the immune cells toward an immunosuppressive phenotype (20).

Adenosinergic pathways identified on different cell populations (21) confer immunosuppressive properties to the cells in different physiological tissues (e.g., cornea and human placenta) (22, 23) and in pathological environments, such as the BM niche of MM (12). Indeed, BM resident cells constitutively express a complete set of cell surface ectonucleotidases, which scattered on different cells drive the production of ADO under metabolic stress (e.g., hypoxia) (24, 25). ADO is also believed to modulate communication between mPCs and normal cells, contributing to the immunocompromised state of MM patients (26).

ADO is produced from the catabolism of mono- and di-nucleotides of adenine (ATP, NAD^+^ and cAMP) (Figure 1). The canonical pathway of ADO production starts from extracellular ATP, which is first hydrolyzed to adenosine diphosphate (ADP) and then to adenosine monophosphate (AMP) by nucleoside triphosphate diphosphohydrolase (NTPDase-1/CD39) or directly by the low-affinity nucleotide pyrophosphatase/phosphodiesterase (NPP/CD203a). The final phosphate group from AMP is cleaved by the 5′-nucleotidase (5′-NT/CD73), thus generating ADO (27). As it is the primary substrate for ectonucleotidases to generate immunosuppressive ADO, ATP implicates the canonical CD39/CD73 tandem in the inception of an anergic tumor milieu (28–30). However, there are some doubts about the ability of this classical pathway to function in closed systems (e.g., BM niche) *in vivo*. For example, the optimal pH for the CD39 enzyme is in the alkaline range of 8–8.3 (31). This might preclude the enzymatic activity of CD39 in a hypoxic TME, where an acidic pH is secondary to lactic acidosis (32). Furthermore, the conversion of extracellular ATP to ADO as catalyzed by CD39 is kinetically complex, with the upstream ADP metabolite generated at high ATP levels of the TME, acting as a feed-forward inhibitor of CD73 (33).

Also, the fact that CD203a has a lower affinity for its substrate ATP than CD39 supports the idea that the alternative ectoenzymatic CD38/CD203a tandem using NAD^+^ as substrate (Figure 1) may become a relevant producer of AMP for ADO production in the BM niche. Therefore, CD39 may not be the only *in vivo* immune system switch that triggers ADO-mediated immunosuppression (34).

Under physiological conditions, the extracellular breakdown of ATP follows the conventional ATP/ADP/AMP/ADO adenosinergic pathway. However, under pathological conditions, the high ATP concentration in the TME causes AMP deaminase (AMPD) to convert AMP into inosine monophosphate (IMP), which in turn is dephosphorylated by 5′-NT/CD73 into inosine (INO) (35) (Figure 1). The IMP pathway (ATP/AMP/IMP/INO), originally thought to be found mainly in the cytosolic cell compartment (36), was recently detected by our group in BM plasma from MM and neuroblastoma patients (3). There are other, alternative(s) substrates (i.e., NAD^+^, cAMP) for the ADO-generating axis in the MM niche (Figure 1). Using T cell leukemia as a model, we confirmed that the canonical CD39/CD73 pathway is flanked by another set of surface molecules leading to the production of ADO, but using NAD^+^ as a leading substrate (9). Components of this alternative pathway are NAD^+^-glycohydrolase/CD38, the ectonucleotide pyrophosphatase/phosphodiesterase 1 (NPP1)/CD203a and the 5′-ectonucleotidase (5′NT)/CD73.

CD38, a transmembrane glycoprotein that lacks an internal signaling domain, is a surface molecule expressed by normal T, B, NK and myeloid populations as well as by different tumor cells (37). The molecule was initially considered as an adhesion/receptor structure, but a review of the evidence suggests that CD38 is not merely a receptor marker (38, 39). Instead, it possesses a number of enzymatic activities ruling NAD^+^ levels inside the BM niche where the mPC grows (25, 40). Indeed, CD38 is located on the mPC surface as well as adjacent non-tumor cells catalyzing the conversion of NAD^+^ to cyclic adenosine diphosphate ribose (cADPR) via cyclase activity and cADPR to ADPR via hydrolase activity (37). ADPR is further hydrolyzed by CD203a to produce AMP. CD203a was recently proposed as a key ectoenzyme because of its ability to convert both ADPR and ATP to AMP, which is subsequently metabolized by CD73 into ADO. Alternatively, a CD73-surrogated ectoenzyme, a Tartrate-Resistant Acid Phosphatase (TRAP), is also functionally active according to the environmental pH (7) (Figure 1).

As can be seen in Figure 2, NAD^+^ relies on the CD38/CD203a tandem and CD73 ectonucleotidase to activate a discontinuous multicellular pathway for ADO production, as detected in plasma aspirates from myeloma BM (12). It is not completely clear whether the alternative CD38/CD203a/CD73 and the canonical CD39/CD73 pathways function cooperatively or whether the relative expression of ectonucleotidases determines which pathway is more active in the hypoxic BM niche. What it sure is that metabolic reprogramming in the BM niche leads to an acidic TME. It is therefore reasonable to believe that the CD38-dependent pathway has a compensatory role for CD39 activity in a BM acidic milieu.

The cyclic nucleotide cAMP signaling pathway is a third alternative route to the production of extracellular ADO (Figure 1). This axis hinges on the cAMP nucleotide-metabolizing membrane-ectoenzyme phosphodiesterase (PDE) and CD73 (41) and it may flank or synergize the known ATP/NAD^+^-catabolic pathways. The cAMP substrate, one of the oldest signaling molecules known, is produced from ATP by membrane-bound adenylyl cyclases (AC) (42, 43). The acidic BM niche improves the egress of cAMP via MRP4 (44) and cAMP efflux might regulate extracellular ADO levels and thus optimize the autocrine and paracrine immunosuppressive effects of ADO. In fact, ADO is rapidly taken up by the red blood cells, which limits its half-life to <1 s in the TME, whereas cAMP is stable in biological fluids, making it possible for it to act at distant sites (45).

ADO levels in the TME are enzymatically balanced by (i) adenosine deaminase (ADA) (which converts ADO into INO) and (ii) ADO kinase (which forms AMP from ADO) (Figure 1). Extracellular ADO homeostasis is also maintained by bidirectional transport through equilibrative nucleoside transporters (ENTs) located in the plasma membrane (46).

The occurrence of an event promoting the extracellular accumulation of nucleotides in MM, their sequential degradation to AMP and the subsequent formation of ADO is followed by a cAMP second messenger pathway coupled to ADO receptors (ADORs) (Figure 1). Indeed, the extracellular accumulation of ADO mediates signals by binding to P1-G protein-coupled purinergic (A1, A2A, A2B, and A3) ADORs expressed by different cells, including immune effectors (47).

## Is There a Link Between ADO Levels and Disease Progression?

ADO is produced by interactions between mPCs and other cells lining the BM niche (25) (Figure 2). This finding suggested that the expression of ectonucleotidases must somehow be linked to the production of immunosuppressive ADO in the BM plasmatic fluid of MM patients to create a protective TME. The different ADO levels in the BM plasma samples analyzed likely reflect (i) variability in the number of ectoenzymes and their activities according to environmental pH (12) or (ii) their tendency to be shed in the biological fluids (48). Further, (iii) several of these molecules are genetically polymorphic, which influences their function. For instance, the expression of CD38 is regulated by a single nucleotide polymorphism (SNP) located in intron 1 (rs6449182; C>G variation) (49).

The accumulation of ADO (>25 μM) in the BM niche via the CD38/CD203a/CD73 and CD39/CD73 axes works sequentially through mPCs/BMSCs/OCs interactions (26). High levels of ADO as determined by cAMP production have a potent stimulatory action on interleukin-6 (IL-6) secretion by BMSCs (50, 51). Because IL-6 is important for normal OB function, the targeting of IL-6 signal pathways may alter the balance between bone resorption and formation. For this reason, mPC and OB interactions in the BM niche contributes to the development of osteolytic lesions in MM (52). IL-6 is also involved in mPC proliferation, survival and disease progression (52, 53). Accordingly, IL-6 correlates with (i) a decrease in serum albumin secondary to increased albuminuria (54) and (ii) up-regulation of factor HIF-1α (16), which parallels MM progression.

Microvesicles (MVs) isolated from the BM plasma of MM patients represent another source of ADO (26). MVs from MM patients express higher levels of adenosinergic ectonucleotidases than those isolated from MGUS/SMM (26). Similarly, the production of ADO was higher when challenging MVs from MM patients than from asymptomatic MGUS/SMM patient samples. A likely explanation is that MVs contribute to the production of ADO in the BM niche (55) (Figure 2).

Results of a recent study in MM patients, evaluated according to the International Staging System (ISS) (56, 57), revealed that ADO levels in BM plasma samples at diagnosis were higher in patients at an advanced stage (ISS = III) with symptomatic MM than in those at the earlier MGUS/SMM stages (pooled ISS = I-II) (12). These findings confirm that ADO production in the BM niche correlates with disease progression and may be useful as a prognostic marker for ISS staging alongside other markers, such as (increasing) serum beta2-microglobulin and (decreasing) serum albumin (58).

These observations support the view that (i) the expression of ectonucleotidases is linked to the production of ADO in the BM plasmatic fluid of MM patients and (ii) that metabolic reprogramming may allow mPCs to construct a microenvironment that favors their survival and protects them from the host immune system. Moreover, the adenosinergic metabolic strategy assists in silencing the immune effectors during progression of MM from indolent monoclonal gammopathy to symptomatic overt disease. It is possible that these observations are only correlative and merely a reflection of tumor burden. Nonetheless, ADO levels in the BM plasma provide a sensitive marker of myeloma progression.

## Impact of Metabolic Reprogramming on Immune Cell Functions

mPCs alter the BM niche, affecting bone cells [e.g., they increase the number and activities of osteoclasts (OCs) and decrease the same on osteoblasts (OBs)], either mediated by soluble factors or by cell-to-cell contacts (6, 59). At the same time, mPCs affect immune events by creating a permissive niche that fosters the colonization of mPCs (60, 61).

Immune cells have regulatory functions originally intended to protect vital organs from inflammatory damage and tumor development (11). However, even activated immune effector T cells that potentially recognize specific tumor-associated antigens have a hard time surviving in the TME while trying to perform their expected functions in harsh metabolic conditions (62). Different kinds of immune cells have developed varied strategies for surviving in the conditions of lactic acidosis (pH ≤ 6.5) created by dysregulated metabolism. Lactic acid is reported as modulating proliferation and activation of human T cells. Indeed, T lymphocytes treated with lactic acid show diminished TCR-mediated activation and trafficking to the TME (63). In addition, effector T cell functions in MM patients are blunted, resulting in paresis of cellular and humoral immunities (64, 65). Tumor cells hijack macrophages (TAMs) via lactic acid (66) and natural killer (NK) cells lose almost all of their functions and reach a state of anergy when exposed to an acidic pH (67). In contrast, myeloid-derived suppressor cells (MDSCs) and regulatory T (Treg) lymphocytes boost tumor growth in acidic conditions (68). The concentrations of lactic acid observed in pathology also inhibit the maturation of dendritic cells (DCs) and their antigen presentation (69). All these conditions in the immune compartment correlate *in vivo* with tumor progression and metastatic spread. Finally, lactic acid causes a reduction in LDH-A expression, which is paralleled by diminished tumor growth and a decline in the number of MDSCs (70). An implication of this is that lactic acid is an immuno-modulatory molecule that can strongly repress anti-tumor immunity.

Within such a scenario, metabolic reprogramming has multiple effects, including the extracellular accumulation of nucleotides (ATP, NAD^+^, cAMP) and of ADO, its main catabolic product (25, 71). From the operational point of view, ADO ligation of ADORA2A (dominantly expressed by most immune cells) is followed by decreased proliferation and by inhibition of the cytolytic anti-tumor activities of cytotoxic T lymphocytes (27, 30, 72), and inhibition of cytotoxicity and IFN-γ release by NK cells (73). These effects are followed by suppression of pro-inflammatory activities and by an increased number of immunoregulatory cells. The outcome is the establishment of a long-lasting immunosuppressive environment (74).

The partial block of ADORA2A may increase the concentration of extracellular AMP, favoring internalization and accumulation of the mononucleotide inside the cell. This would lead to activation of the AMP-dependent protein kinase (AMPK) (75), inducing a positive effect on the AMPK/mTOR/p70S6K/rpS6 protein axis, which is reported as inducing suppression of T cell proliferation in human melanoma cells through an adenosinergic pathway led by CD38 (76). The high levels of ADO measured in the culture supernatants of primary melanoma cells and the BM plasma from MM patients (12) were also detected by metabolomic screening using AICAR (5-Aminoimidazole-4-carboxamide ribonucleotide)-treated malignant cells identifying pyrimidine starvation as the mechanism of AICAR-induced apoptosis in mPCs (77). AICAR is a metabolic intermediate in the enzymatic conversion of AMP into inosine monophosphate (IMP), catalyzed by ADO deaminase (3). As an analog of AMP, AICAR activates AMP-dependent protein kinase (AMPK) activity, a signal molecule reported as a potential target in MM that induces G1 arrest in mPCs (78). High extracellular ADO levels, by ligation of low affinity ADORA2B, can influence the antigen-presenting activity of DCs (79, 80) and activate normal infiltrating cells that block the anti-tumor immune response (such as Tregs, MDSCs, and TAMs) (81), leading to peripheral tolerance (Figure 2). Although these cell subsets are recruited to the tumor site to fine-tune immune activation, they have the perverse effect of boosting tumor growth (28).

## Therapeutic Strategies to Counteract Adenosine Suppressive Mechanisms in MM

### Immune Checkpoint Molecules in MM Bone Marrow

**Figure 3:**
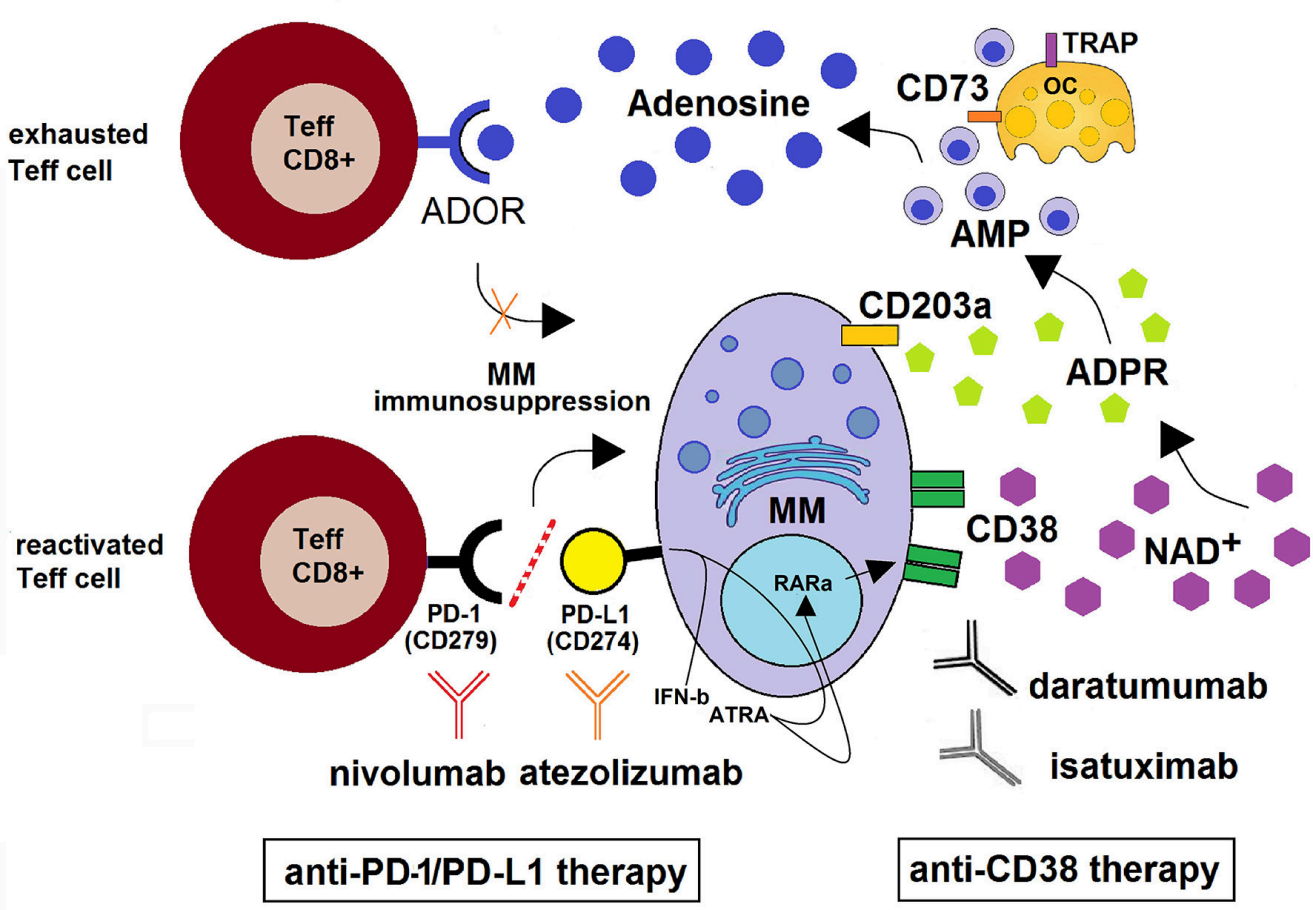
Schematic depiction of the CD38/CD203a/CD73 adenosinergic pathway as a major mechanism of the acquired resistance to anti-PD-1/PD-L1 blockade within the BM niche. CD38^+high^ MM cells catalyze NAD^+^ transformation to ADO via the CD38/CD203a/CD73 ectoenzymatic pathway, discontinuously expressed by BM resident cells (MM, OCs, OBs, BMSC). This step is followed by (i) the activation of the ADOR A2A and A2B on cytotoxic T-lymphocytes, with suppression of their anti-tumor functions and (ii) the induction of an anti-PD-1/PD-L1-mediated resistance to the increase of cytotoxic T-cell infiltration in the BM niche. Anti–PD-1/PD-L1-resistant MM cells also produce soluble mediators (such as IFN-β and ATRA) leading to increased expression of CD38 on mPCs via RARα. This mechanism support the use of anti-CD38 mAbs (e.g., daratumumab and isatuximab) with the ability to inhibit CD38 cyclase activity. When used in combination with PD-1/PD-L1 immune checkpoint blockade (e.g., nivolumab and atezolizumab), the result may be an improvement of antitumor immune responses with reactivation of CD8^+^ T effector lymphocytes leading to a control of MM cells.

Preclinical and clinical studies revealed that most tumors overexpress immune checkpoint (ICP) molecules, of which the most studied are programmed death-1 (PD-1) and its ligand (PD-L1) and cytotoxic T-lymphocyte-associated protein-4 (CTLA-4) (82, 83). The activation of ICP rules anergy, apoptosis and “exhaustion” to initiate T cell suppression (84–86). In a similar fashion, mPCs can evade immunosurveillance by means of multiple mechanisms of immunosuppression (74), such as the ability of mPCs to hijack inhibitory ICP suppressive mechanisms (87). Therapeutic strategies incorporating inhibition of ICP have shown promising results, although high rates of resistance limit their efficacy (88). For instance, a large subset of patients still remains refractory following PD-1/PD-L1 ICP blockade (89, 90). Recently, the purinergic pathway promoting ADO generation through the CD38/CD203a/CD73 pathway (9) was identified as the main obstacle to the therapeutic benefit of anti–PD-1/PD-L1 blockade (91, 92). The same studies also determined that tumors treated with PD-1/PD-L1-monoclonal antibodies (mAbs) endowed with blocking properties developed resistance through the metabolic upregulation of CD38, akin to that induced by all-trans retinoic acid (ATRA) and interferon-β in the TME (as in non-small cell lung cancer, NSCLC). A pre-existing and inducible high expression of CD38 (as in mPCs) is thought to be the main hindrance to the therapeutic potential of the anti–PD-1/PD-L1 blockade (Figure 3). Indeed, it has been reported that (i) CD38 inhibits CD8^+^ T-cell functions via ADO receptor signaling (76), while either (ii) inhibiting CD38 or blocking ADO receptors was effective in overcoming resistance to combined ICP immunotherapeutic strategies (92). Further, (iii) CD38 KO tumors grow much more slowly than CD38^+^ wild-type tumors in wild-type mice (92). However, the protective effect of CD38 vanishes in the absence of CD8^+^ T cells, suggesting that CD38-expressing cells impair CD8^+^ T cell functions. Together, these data indicate that the NAD^+^ adenosinergic pathway helps sustain the production of immunosuppressant ADO in the modified adaptive immune response to anti-PD-1/PD-L1 treatment (Figure 3).

The endpoint of ADO signaling is the induction of “anergic” effector cells, which suggests that extracellular ADO functions as a negative ICP molecule (93). This hypothesis is strengthened by evidence of synergic anti-tumor effects elicited by combining anti-PD-1/CTLA-4 and inhibitors of ADO production or signaling (94).

### Reducing CD38 Surface Levels

CD38 is expressed at different levels on mPCs from all MM patients (95, 96). A decrease in the level of CD38 in MM cells can occur in several ways: (i) treatment with anti-CD38 mAbs; (ii) generation of MVs followed by their uptake of the CD38-mAb complexes by FcR^+^-expressing cells; and (iii) trogocytosis (97). It has been observed that anti-CD38 mAbs (e.g., daratumumab) ligation on mPCs is followed by the aggregation, polarization and release of MVs derived from cell membranes and expressing adenosinergic molecules (CD39, CD203a, CD73) clustered in lipid domains. MVs isolated from the BM plasma of MM patients also contain the target CD38 as well as the specific monoclonal IgG (98). While the exact fate of the MVs is unknown, MV bearing monoclonal IgG exit the BM niche and cluster around cells expressing FcR. Since MVs fuse with the target cells, modulation of immune responses is expected (55).

Transfer of CD38 from the MM cell surface to effector cells either by trogocytic transfer or vesiculation might compromise therapeutic efficacy because of a reduction in mAbs that eliminate MM cells via CDC and ADCC (97, 99). This reduction in surface CD38 could have several beneficial effects. Firstly, CD38 is an immunomodulatory molecule that inhibits T-cell functions via ADOR signaling (100). It is thus possible that a simultaneous down-regulation of CD38 (and associated adenosinergic molecules) on MM cells as well as in TME cells by trogocytosis and MV formation may lead to (i) decreased levels of ADO in the BM niche (12) and, consequently, (ii) fewer immune-related adverse events associated with ICP blockade (90). In fact, as shown in Figure 3, treatment with anti-CD38 mAbs in combination with anti-PD-1/PD-L1 may induce expansion of BM T effector cells in MM patients.

CD38 can also be transferred by trogocytosis to effector T lymphocytes, along with other molecules located on CD38^+^mPCs membrane domain. For instance, the expression of adhesion proteins on the surface of (i) MM cells (e.g., CD56, CD49d, and CD138) (101) or of (ii) non-immune and endothelial cells (e.g., CD31/PECAM1) (102) resident in the BM niche are transferred, resulting in diminished expression. It is therefore likely that CD38^+^mPC interaction with CD31^+^BMSCs in the protective myeloma niche is hindered, leading to a reduction in pro-survival signals (103).

In contrast with conventional tumor therapies, mAb immunotherapy targets the immune response to provoke a systemic anti-tumor response (104, 105). CD38 is a valuable target for therapeutic mAbs because of (i) its ability to impair tumor growth either by directly targeting cells or by inducing immune modulation (106). Other significant advantages provided by mAbs are (ii) the successful induction of durable responses and increased survival in various types of cancer (107–109).

Pharma companies recognized the value of CD38 as an ideal target for treating human MM with mAbs because of its favorable expression during ontogenesis (37, 110). Indeed, CD38 is not expressed by early hematological precursors (111) and CD38 expression is maintained in spite of the genomic differences marking mPCs.

In the BM niche, NAD^+^ metabolization is mediated by CD38 and elicits rapid functional responses leading to significant accumulation of ADO that induces immune silencing. It is thus reasonable to assume that a mAb-mediated reduction of CD38 on mPCs, mediated by the uptake of CD38^+^MVs-mAb complexes by FcR^+^-immune cells, may contribute to an improved host-antitumor immune response (112). Two of the available anti-CD38 therapeutic mAbs, daratumumab and isatuximab, modulate the enzymatic activity of CD38 *in vitro*, which is able to reduce immunosuppressive ADO levels (3). Daratumumab (human IgG1k), the first therapeutic mAb approved *in vivo* (113, 114), inhibits CD38 cyclase activity, while enhancing hydrolase enzymatic activity (115). Its ligand effect on NAD^+^ catalysis was determined on mPCs isolated from the BM plasma of MM patients and, for comparative evaluation, on a continuous human myeloma cell line. It was seen that daratumumab (100 μg/mL) inhibits the cyclase activity of CD38 *in vitro* (45% ± 5 and 32% ± 10, respectively). Furthermore, daratumumab ligation is followed by increased hydrolysis of cADPR (20% ± 5). Thus, daratumumab modulates the enzymatic activities of CD38, partially dampening cyclase activity, while simultaneously enhancing hydrolase activity. These results were measured by HPLC chromatography tests using NAD^+^ (or the surrogate NGD^+^) as the substrate for cyclase and, cADPR for hydrolase (12, 25). So far, the results indicate that NAD^+^ (and NGD^+^) are decreased by consumption secondary to CD38 catalysis and cADPR is also reduced. In contrast, ADPR is increased in the presence of daratumumab. Reduced cADPR levels may lead to reduced Ca^2+^ mobilization, which decreases signaling potential. Increased ADPR levels, which contribute to adenosinergic immune suppression, add a further element of complexity to the context of NAD^+^ homeostasis and tumor survival in closed systems, (e.g., the BM niche). It is still worthwhile to evaluate the specific contribution of each CD38 cyclase/hydrolase mechanism to the clinical features of MM. In addition, daratumumab-mediated reduction of CD38 on MM cells may also decrease the generation of immunosuppressive ADO molecules (12, 26), which would result in an improved host-anti-tumor immune response (91). Further investigation is needed to determine whether anti-CD38 *in vivo* therapy also modulates the enzymatic activities of the molecule. However, on the basis of *in vitro* experimental observations, we hypothesize that specific monoclonal IgG1 antibodies (e.g., daratumumab and isatuximab) might modulate both the cyclase and hydrolase enzymatic activities of CD38 *in vivo*. This hypothesis is supported by initial experimental evaluation of ADO in paired blood and BM plasma samples from MM patients, obtained before and after treatment with daratumumab (in collaboration with Dr. van de Donk, Department of Hematology, VU University Medical Center, Amsterdam, The Netherlands). Indeed, the experimental adenosinergic trend observed (e.g., decay of ADO contents after daratumumab treatment) seem consistent with the *in vitro* counterpart (Horenstein, personal communication).

## Mechanisms of Resistance to Immunotherapy: mABs and Immunomodulator Drugs

Increased understanding of the interactions between malignant cells and the immune system has paved the way to immunotherapy for cancer patients (116). Despite some favorable outcomes, most patients do not respond, likely because of intrinsic tumor resistance mechanisms. These include (i) decreased or absent antigen expression [e.g., tumor antigens, MHC I receptors, MHC I chain-related gene A and B (MICA and MICB)]; (ii) changes in the expression of cell receptors (e.g., tumor-expressed markers, PD-1/PD-L1, CTLA-4, among other ICPs); and (iii) alterations in cellular enzymes and metabolic pathways [e.g., CD38/NAD^+^glycohydrolase; indoleamine 2,3-dioxygenase (IDO)] (37, 117) are the most likely involved in changes within the TME, resulting in a lack of response to immunotherapy (109).

Absent of MICA antigen expression has been suggested as a potential predictor of the efficacy of future immunotherapies using cytokine-induced killer (CIK) cells, a T cell population obtained by *in vitro* differentiation of peripheral blood mononuclear cells (PBMC) that represent a promising immunological approach in cancer (118). It has been shown that CIK cells are able (i) to produce extracellular ADO via canonical (CD39/CD73) and/or alternative (CD38/CD203a/CD73 or CD203a/CD73) pathways (119) and (ii) to modulate these ectonucleotidases during PBMC to CIK differentiation. This means that it may be possible to modulate ADO-generating ectoenzymes pharmacologically to improve CIK cell performance. Other treatments for enhancing MICA expression in MM myeloma cell lines and increase cytotoxicity are also being explored (120).

Immunotherapy protocols have also revealed that the initial benefits of mAb therapy can be followed by resistance to anti-tumor immune responses (88). The mechanisms of resistance might be secondary to reprogrammed metabolism, which generates immune privileges in the TME. Such dysregulated metabolic conditions may influence the deterioration of the mAb, reducing its therapeutic efficacy (121–123). There are several possible explanations for such deterioration (e.g., fragmentation, aggregation or denaturation) and potential loss of mAb activity. One is related to the acidic extracellular pH observed in the TME. These effects depend on the properties of the individual mAb as well as on the environmental characteristics where the mAb is expected to operate (124). Furthermore, an acidic pH may induce degradation of the aspartate amino acid in the complementarity-determining regions (CDR): this may reduce or influence the ability of the mAb to bind to its epitope (123). Therefore, the highly acidic nature of the TME in the MM niche is of extreme relevance in determining the therapeutic activity of the anti-CD38 mAb selected.

Although anti-CD38/daratumumab-mediated therapy has single-agent efficacy in MM disease, clinical trials have suggested that outcomes are improved when treatment is combined with immunomodulator drugs (IMiDs: e.g., dexamethasone, thalidomide, doxorubicin, lenalidomide, among others) (125, 126). It must be kept in mind that the positive charge acquired by the weak chemical base (i.e., doxorubicin) in an acidic BM environment inhibits its permeability across biological membranes. Consequently, the efficacy of drug delivery and the resistance mechanisms are now postulated to link an acidic TME with the dynamics of the tumor cell membrane. Importantly, additional mechanisms of resistance continue to be discovered, further elucidating the complex interactions between malignant cells and the immune system (88).

## Conclusions

An accurate depiction of the metabolism of extracellular nucleotides facilitates the design of original strategies for inactivating ADO-dependent immunosuppressive mechanisms. Because ICP therapies, such as anti-PD-1/PD-L1, have acquired resistance to CD38-generated ADO, pathways driven by CD38 that involve ADO production may be considered as a promising therapeutic approach. In line with this, several options to counteract the immunosuppressive effects of ADO are currently under analysis (93, 125). Synergic strategies being evaluated include (i) inhibition of nucleotide-release channels, (ii) use of inhibitors of ADO generation by the CD39/CD73 and CD38/CD203a/CD73 ectoenzymatic pathways, (iii) use of drugs degrading extracellular ADO. Further approaches are (iv) the use of A2A and A2B ADOR antagonists. Still other potential strategies rely on (v) inhibitors of hypoxia-HIF-1α signaling, (vi) activatory mechanisms of ADO hydrolytic deamination to INO [a caveat is that it can mediate immunosuppressive effects long after ADO catabolization (35)], and to (vii) AMP synthesis from ADO by ADO kinase (127). These are the main options under consideration today. Future studies seek additional targets that might amplify the antitumor immune response, with the aim of increasing the rate of lasting response to immunotherapy. For instance, AMPD is a purine metabolic enzyme that converts AMP to IMP (see section Adenosine Production within the BM Niche). The enzyme was analyzed in hematological malignancies to investigate whether it is suitable as a novel target for MM therapy (128). The report raised the possibility that AMPD inhibition might be useful as a novel therapeutic strategy for MM. Moreover, AMPD inhibitors induced cell death in myeloma cell lines.

One of the aims of the present review has been to provide support for the view that the metabolism/immunity tandem can be useful in the development of a new generation of MM therapies. Some of the proposals mentioned are now entering into clinical trials. The results will validate the efficacy of treatment in terms of its impact on disease progression. A precise definition of the mechanisms through which the intricate purinome network operates in MM will facilitate the design of predictive diagnostic procedures as well as the adoption of pharmacological agents able to target adenosinergic pathways. Along with drugs directly targeting mPCs, these results are expected to lead to future theragnostic applications.

## Author Contributions

AH and FM analyzed data from the literature and wrote the manuscript. CB contributed to unpublished experiments. FMo contributed to analyzed data from the literature.

### Conflict of Interest Statement

FM has received research support from Janssen Pharmaceutical, Celgene, and Tusk Therapeutics. Advisory Board and delivered lectures for Janssen, Takeda, and Sanofi. The remaining authors declare that the research was conducted in the absence of any commercial or financial relationships that could be construed as a potential conflict of interest. The reviewer SD declared a shared affiliation, though no other collaboration, with several of the authors AH, CB, FM, and FMo to the handling Editor.

## References

[1] Palumbo A Anderson K. Multiple myeloma. N Engl J Med. (2011) 364:1046–60. 10.1056/NEJMra1011442 21410373

[2] Bianchi G Munshi NC. Pathogenesis beyond the cancer clone(s) in multiple myeloma. Blood. (2015) 125:3049–58. 10.1182/blood-2014-11-568881 25838343 PMC4432002

[3] Horenstein AL Morandi F Bracci C Pistoia V Malavasi F. Functional insights into nucleotide-metabolizing ectoenzymes expressed by bone marrow-resident cells in patients with multiple myeloma. Immunol Lett. (2018) 205:40–50. 10.1016/j.imlet.2018.11.007 30447309

[4] Lukashev D Ohta A Sitkovsky M. Hypoxia-dependent anti-inflammatory pathways in protection of cancerous tissues. Cancer Metastasis Rev. (2007). 26:273–9. 10.1007/s10555-007-9054-2 17404693

[5] Pezzolo A Marimpietri D Raffaghello L Cocco C Pistorio A Gambini C. Failure of anti tumor-derived endothelial cell immunotherapy depends on augmentation of tumor hypoxia. Oncotarget. (2014) 5:10368–81. 10.18632/oncotarget.2015 25362644 PMC4279379

[6] Abe M Hiura K Wilde J Shioyasono A Moriyama K Hashimoto T. Osteoclasts enhance myeloma cell growth and survival via cell-cell contact: a vicious cycle between bone destruction and myeloma expansion. Blood. (2004) 104:2484–91. 10.1182/blood-2003-11-3839 15187021

[7] Quarona V Ferri V Chillemi A Bolzoni M Mancini C Zaccarello G. Unraveling the contribution of ectoenzymes to myeloma life and survival in the bone marrow niche. Ann N Y Acad Sci. (2015) 1335:10–22. 10.1111/nyas.12485 25048519

[8] Apasov S Koshiba M Redegeld F Sitkovsky MV. Role of extracellular ATP and P1 and P2 classes of purinergic receptors in T-cell development and cytotoxic T lymphocyte effector functions. Immunol Rev. (1995) 146:5–19. 10.1111/j.1600-065X.1995.tb00680.x 7493760

[9] Horenstein AL Chillemi A Zaccarello G Bruzzone S Quarona V Zito A. A CD38/CD203a/CD73 ectoenzymatic pathway independent of CD39 drives a novel adenosinergic loop in human T lymphocytes. Oncoimmunology. (2013) 2:e26246. 10.4161/onci.26246 24319640 PMC3850273

[10] Allard D Turcotte M Stagg J. Targeting A2 adenosine receptors in cancer. Immunol Cell Biol. (2017) 95:333–9. 10.1038/icb.2017.8 28174424

[11] Sitkovsky MV Ohta A. The ‘danger' sensors that STOP the immune response: the A2 adenosine receptors? Trends Immunol. (2005) 26:299–304. 10.1016/j.it.2005.04.004 15922945

[12] Horenstein AL Quarona V Toscani D Costa F Chillemi A Pistoia V. Adenosine generated in the bone marrow niche through a CD38-mediated pathway correlates with progression of human myeloma. Mol Med. (2016) 22:694–704. 10.2119/molmed.2016.00198 27761584 PMC5135080

[13] Kuehl WM Bergsagel PL. Molecular pathogenesis of multiple myeloma and its premalignant precursor. J Clin Invest. (2012) 122:3456–63. 10.1172/JCI61188 23023717 PMC3461901

[14] Manier S Kawano Y Bianchi G Roccaro AM Ghobrial IM. Cell autonomous and microenvironmental regulation of tumor progression in precursor states of multiple myeloma. Curr Opin Hematol. (2016) 23:426–33. 10.1097/MOH.0000000000000259 27101529

[15] Volonte C D'Ambrosi N. Membrane compartments and purinergic signalling: the purinome, a complex interplay among ligands, degrading enzymes, receptors and transporters. FEBS J. (2009) 276:318–29. 10.1111/j.1742-4658.2008.06793.x 19076212

[16] Semenza GL. HIF-1 mediates metabolic responses to intratumoral hypoxia and oncogenic mutations. J Clin Invest. (2013) 123:3664–71. 10.1172/JCI67230 23999440 PMC3754249

[17] Hanahan D Weinberg RA. Hallmarks of cancer: the next generation. Cell. (2011) 144:646–74. 10.1016/j.cell.2011.02.013 21376230

[18] Keibler MA Wasylenko TM Kelleher JK Iliopoulos O Vander Heiden MG Stephanopoulos G. Metabolic requirements for cancer cell proliferation. Cancer Metab. (2016) 4:16. 10.1186/s40170-016-0156-6 27540483 PMC4989334

[19] Hsu PP Sabatini DM. Cancer cell metabolism: Warburg and beyond. Cell. (2008) 134:703–7. 10.1016/j.cell.2008.08.021 18775299

[20] Bolzoni M Toscani D Costa F Vicario E Aversa F Giuliani N. The link between bone microenvironment and immune cells in multiple myeloma: Emerging role of CD38. Immunol Lett. (2018) 205:65–70. 10.1016/j.imlet.2018.04.007 29702149

[21] Ferretti E Horenstein AL Canzonetta C Costa F Morandi F. Canonical and non-canonical adenosinergic pathways. Immunol Lett. (2018) 205:25–30. 10.1016/j.imlet.2018.03.007 29550257

[22] Horenstein AL Sizzano F Lusso R Besso FG Ferrero E Deaglio S. CD38 and CD157 ectoenzymes mark cell subsets in the human corneal limbus. Mol Med. (2009) 15:76–84. 10.2119/molmed.2008.00108 19052657 PMC2593003

[23] Cecati M Emanuelli M Giannubilo SR Quarona V Senetta R Malavasi F. Contribution of adenosine-producing ectoenzymes to the mechanisms underlying the mitigation of maternal-fetal conflicts. J Biol Regul Homeost Agents. (2013) 27:519–29. 23830401

[24] Sitkovsky MV Kjaergaard J Lukashev D Ohta A. Hypoxia-adenosinergic immunosuppression: tumor protection by T regulatory cells and cancerous tissue hypoxia. Clin Cancer Res. (2008) 14:5947–52. 10.1158/1078-0432.CCR-08-0229 18829471

[25] Horenstein AL Chillemi A Quarona V Zito A Roato I Morandi F. NAD^+^-Metabolizing ectoenzymes in remodeling tumor-host interactions: the human myeloma model. Cells. (2015) 4:520–37. 10.3390/cells4030520 26393653 PMC4588049

[26] Chillemi A Quarona V Antonioli L Ferrari D Horenstein AL Malavasi F. Roles and modalities of ectonucleotidases in remodeling the multiple myeloma niche. Front Immunol. (2017) 8:305. 10.3389/fimmu.2017.00305 28373875 PMC5357780

[27] Stagg J Smyth MJ. Extracellular adenosine triphosphate and adenosine in cancer. Oncogene. (2010) 29:5346–58. 10.1038/onc.2010.292 20661219

[28] Stagg J Johnstone RW Smyth MJ. From cancer immunosurveillance to cancer immunotherapy. Immunol Rev. (2007) 220:82–101. 10.1111/j.1600-065X.2007.00566.x 17979841

[29] Serra S Horenstein AL Vaisitti T Brusa D Rossi D Laurenti L. CD73-generated extracellular adenosine in chronic lymphocytic leukemia creates local conditions counteracting drug-induced cell death. Blood. (2011) 118:6141–52. 10.1182/blood-2011-08-374728 21998208 PMC3342854

[30] Sitkovsky MV Hatfield S Abbott R Belikoff B Lukashev D Ohta A. Hostile, hypoxia-A2-adenosinergic tumor biology as the next barrier to overcome for tumor immunologists. Cancer Immunol Res. (2014) 2:598–605. 10.1158/2326-6066.CIR-14-0075 24990240 PMC4331061

[31] Milosevic M Petrovic S Velickovic N Grkovic I Ignjatovic M Horvat A. ATP and ADP hydrolysis in cell membranes from rat myometrium. Mol Cell Biochem. (2012) 371:199–208. 10.1007/s11010-012-1436-2 22956447

[32] Jiang B. Aerobic glycolysis and high level of lactate in cancer metabolism and microenvironment. Genes Dis. (2017) 4:25–7. 10.1016/j.gendis.2017.02.003 30258905 PMC6136593

[33] Gordon EL Pearson JD Slakey LL. The hydrolysis of extracellular adenine nucleotides by cultured endothelial cells from pig aorta. Feed-forward inhibition of adenosine production at the cell surface. J Biol Chem. (1986) 261:15496–507. 3023320

[34] Zhao H Bo C Kang Y Li H. What else can CD39 tell us? Front Immunol. (2017) 8:727. 10.3389/fimmu.2017.00727 28690614 PMC5479880

[35] Yuan S Jiang X Zhou X Zhang Y Teng T Xie P. Inosine alleviates depression-like behavior and increases the activity of the ERK-CREB signaling in adolescent male rats. Neuroreport. (2018) 29:1223–9. 10.1097/WNR.0000000000001101 30028377

[36] Barsotti C Ipata PL. Metabolic regulation of ATP breakdown and of adenosine production in rat brain extracts. Int J Biochem Cell Biol. (2004) 36:2214–25. 10.1016/j.biocel.2004.04.015 15313467

[37] Malavasi F Deaglio S Funaro A Ferrero E Horenstein AL Ortolan E. Evolution and function of the ADP ribosyl cyclase/CD38 gene family in physiology and pathology. Physiol Rev. (2008) 88:841–86. 10.1152/physrev.00035.2007 18626062

[38] Malavasi F Funaro A Roggero S Horenstein A Calosso L Mehta K. Human CD38: a glycoprotein in search of a function. Immunol Today. (1994) 15:95–7. 10.1016/0167-5699(94)90148-1 8172650

[39] van de Donk N Richardson PG Malavasi F CD38 antibodies in multiple myeloma: back to the future. Blood. (2018) 131:13–29. 10.1182/blood-2017-06-740944 29118010

[40] Chiarugi A Dolle C Felici R Ziegler M. The NAD metabolome–a key determinant of cancer cell biology. Nat Rev Cancer. (2012) 12:741–52. 10.1038/nrc3340 23018234

[41] Giron MC Bin A Brun P Etteri S Bolego C Florio C. Cyclic AMP in rat ileum: evidence for the presence of an extracellular cyclic AMP-adenosine pathway. Gastroenterology. (2008) 134:1116–26. 10.1053/j.gastro.2008.01.030 18316082

[42] Beavo JA Brunton LL. Cyclic nucleotide research – still expanding after half a century. Nat Rev Mol Cell Biol. (2002) 3:710–8. 10.1038/nrm911 12209131

[43] Pleli T Mondorf A Ferreiros N Thomas D Dvorak K Biondi RM. Activation of adenylyl cyclase causes stimulation of adenosine receptors. Cell Physiol Biochem. (2018) 45:2516–28. 10.1159/000488270 29587249

[44] Hofer AM Lefkimmiatis K. Extracellular calcium and cAMP: second messengers as “third messengers”? Physiology. (2007) 22:320–7. 10.1152/physiol.00019.2007 17928545

[45] Copsel S Garcia C Diez F Vermeulem M Baldi A Bianciotti LG. Multidrug resistance protein 4 (MRP4/ABCC4) regulates cAMP cellular levels and controls human leukemia cell proliferation and differentiation. J Biol Chem. (2011) 286:6979–88. 10.1074/jbc.M110.166868 21205825 PMC3044954

[46] Guida L Bruzzone S Sturla L Franco L Zocchi E De Flora A. Equilibrative and concentrative nucleoside transporters mediate influx of extracellular cyclic ADP-ribose into 3T3 murine fibroblasts. J Biol Chem. (2002) 277:47097–105. 10.1074/jbc.M207793200 12368285

[47] Fredholm BB IJzerman AP Jacobson KA Linden J Müller CE. International union of basic and clinical pharmacology. LXXXI. Nomenclature and classification of adenosine receptors–an update. Pharmacol Rev. (2011) 63:1–34. 10.1124/pr.110.003285 21303899 PMC3061413

[48] Funaro A Horenstein AL Calosso L Morra M Tarocco RP Franco L. Identification and characterization of an active soluble form of human CD38 in normal and pathological fluids. Int Immunol. (1996) 8:1643–50. 10.1093/intimm/8.11.1643 8943558

[49] Ferrero E Saccucci F Malavasi F. The human CD38 gene: polymorphism, CpG island, and linkage to the CD157 (BST-1) gene. Immunogenetics. (1999) 49:597–604. 10.1007/s002510050654 10369916

[50] Kudo O Sabokbar A Pocock A Itonaga I Fujikawa Y Athanasou NA. Interleukin-6 and interleukin-11 support human osteoclast formation by a RANKL-independent mechanism. Bone. (2003) 32:1–7. 10.1016/S8756-3282(02)00915-8 12584029

[51] Evans BA Elford C Pexa A Francis K Hughes AC Deussen A. Human osteoblast precursors produce extracellular adenosine, which modulates their secretion of IL-6 and osteoprotegerin. J Bone Miner Res. (2006) 21:228–36. 10.1359/JBMR.051021 16418778

[52] Hunsucker SA Magarotto V Kuhn DJ Kornblau SM Wang M Weber DM. Blockade of interleukin-6 signalling with siltuximab enhances melphalan cytotoxicity in preclinical models of multiple myeloma. Br J Haematol. (2011) 152:579–92. 10.1111/j.1365-2141.2010.08533.x 21241278 PMC3402914

[53] Burger R. Impact of interleukin-6 in hematological malignancies. Transfus Med Hemother. (2013) 40:336–43. 10.1159/000354194 24273487 PMC3822278

[54] Kasztan M Piwkowska A Kreft E Rogacka D Audzeyenka I Szczepanska-Konkel M. Extracellular purines' action on glomerular albumin permeability in isolated rat glomeruli: insights into the pathogenesis of albuminuria. Am J Physiol Renal Physiol. (2016) 311:F103–11. 10.1152/ajprenal.00567.2015 27076649

[55] Morandi F Marimpietri D Horenstein AL Bolzoni M Toscani D Costa F. Microvesicles released from multiple myeloma cells are equipped with ectoenzymes belonging to canonical and non-canonical adenosinergic pathways and produce adenosine from ATP and NAD. Oncoimmunology. (2018) 7:e1458809. 10.1080/2162402X.2018.1458809 30221054 PMC6136872

[56] Greipp PR San Miguel J Durie BG Crowley JJ Barlogie B Blade J. International staging system for multiple myeloma. J Clin Oncol. (2005) 23:3412–20. 10.1200/JCO.2005.04.242 15809451

[57] Mikhael JR Dingli D Roy V Reeder CB Buadi FK Hayman SR. Management of newly diagnosed symptomatic multiple myeloma: updated Mayo Stratification of Myeloma and Risk-Adapted Therapy (mSMART) consensus guidelines 2013. Mayo Clin Proc. (2013) 88:360–76. 10.1016/j.mayocp.2013.01.019 23541011

[58] Kyrtsonis MC Maltezas D Tzenou T Koulieris E Bradwell AR. Staging systems and prognostic factors as a guide to therapeutic decisions in multiple myeloma. Semin Hematol. (2009) 46:110–7. 10.1053/j.seminhematol.2009.02.004 19389494

[59] An G Acharya C Feng X Wen K Zhong M Zhang L. Osteoclasts promote immune suppressive microenvironment in multiple myeloma: therapeutic implication. Blood. (2016) 128:1590–603. 10.1182/blood-2016-03-707547 27418644 PMC5034739

[60] Costa F Toscani D Chillemi A Quarona V Bolzoni M Marchica V. Expression of CD38 in myeloma bone niche: a rational basis for the use of anti-CD38 immunotherapy to inhibit osteoclast formation. Oncotarget. (2017) 8:56598–611. 10.18632/oncotarget.17896 28915615 PMC5593586

[61] Kawano Y Roccaro AM Ghobrial IM Azzi J. Multiple myeloma and the immune microenvironment. Curr Cancer Drug Targets. (2017) 17:806–18. 10.2174/1568009617666170214102301 28201978

[62] Balkwill FR Capasso M Hagemann T. The tumor microenvironment at a glance. J Cell Sci. (2012) 125(Pt 23):5591–6. 10.1242/jcs.116392 23420197

[63] Cascone T McKenzie JA Mbofung RM Punt S Wang Z Xu C. Increased tumor glycolysis characterizes immune resistance to adoptive t cell therapy. Cell Metab. (2018) 27:977–87.e4. 10.1016/j.cmet.2018.02.024 29628419 PMC5932208

[64] Roodman GD. Pathogenesis of myeloma bone disease. Leukemia. (2009) 23:435–41. 10.1038/leu.2008.336 19039321

[65] Chen DS Mellman I. Oncology meets immunology: the cancer-immunity cycle. Immunity. (2013) 39:1–10. 10.1016/j.immuni.2013.07.012 23890059

[66] Bronte V. Tumor cells hijack macrophages via lactic acid. Immunol Cell Biol. (2014) 92:647–9. 10.1038/icb.2014.67 25091608

[67] Dosani T Carlsten M Maric I Landgren O The cellular immune system in myelomagenesis: NK cells and T cells in the development of myeloma [corrected] and their uses in immunotherapies. Blood Cancer J. (2015) 5:e306 10.1038/bcj.2015.32 25885426 PMC4450330

[68] Gorgun GT Whitehill G Anderson JL Hideshima T Maguire C Laubach J. Tumor-promoting immune-suppressive myeloid-derived suppressor cells in the multiple myeloma microenvironment in humans. Blood. (2013) 121:2975–87. 10.1182/blood-2012-08-448548 23321256 PMC3624943

[69] Gottfried E Kunz-Schughart LA Ebner S Mueller-Klieser W Hoves S Andreesen R. Tumor-derived lactic acid modulates dendritic cell activation and antigen expression. Blood. (2006) 107:2013–21. 10.1182/blood-2005-05-1795 16278308

[70] Brand A Singer K Koehl GE Kolitzus M Schoenhammer G Thiel A. LDHA-associated lactic acid production blunts tumor immunosurveillance by T and NK cells. Cell Metab. (2016) 24:657–71. 10.1016/j.cmet.2016.08.011 27641098

[71] Allard B Beavis PA Darcy PK Stagg J. Immunosuppressive activities of adenosine in cancer. Curr Opin Pharmacol. (2016) 29:7–16. 10.1016/j.coph.2016.04.001 27209048

[72] Ohta A Kini R Ohta A Subramanian M Madasu M Sitkovsky M. The development and immunosuppressive functions of CD4^+^ CD25^+^ FoxP3^+^ regulatory T cells are under influence of the adenosine-A2A adenosine receptor pathway. Front Immunol. (2012) 3:190. 10.3389/fimmu.2012.00190 22783261 PMC3389649

[73] Morandi F Horenstein AL Chillemi A Quarona V Chiesa S Imperatori A. CD56^bright^CD16- NK cells produce adenosine through a CD38-mediated pathway and act as regulatory cells inhibiting autologous CD4^+^ T cell proliferation. J Immunol. (2015) 195:965–72. 10.4049/jimmunol.1500591 26091716

[74] Ohta A. A metabolic immune checkpoint: adenosine in tumor microenvironment. Front Immunol. (2016) 7:109. 10.3389/fimmu.2016.00109 27066002 PMC4809887

[75] Camici M Allegrini S Tozzi MG. Interplay between adenylate metabolizing enzymes and AMP-activated protein kinase. FEBS J. (2018) 285:3337–52. 10.1111/febs.14508 29775996

[76] Morandi F Morandi B Horenstein AL Chillemi A Quarona V Zaccarello G. A non-canonical adenosinergic pathway led by CD38 in human melanoma cells induces suppression of T cell proliferation. Oncotarget. (2015) 6:25602–18. 10.18632/oncotarget.4693 26329660 PMC4694853

[77] Bardeleben C Sharma S Reeve JR Bassilian S Frost P Hoang B. Metabolomics identifies pyrimidine starvation as the mechanism of 5-aminoimidazole-4-carboxamide-1-beta-riboside-induced apoptosis in multiple myeloma cells. Mol Cancer Ther. (2013) 12:1310–21. 10.1158/1535-7163.MCT-12-1042 23585020 PMC3707969

[78] Rattan R Giri S Singh AK Singh I. 5-Aminoimidazole-4-carboxamide-1-beta-D- ribofuranoside inhibits cancer cell proliferation *in vitro* and *in vivo* via AMP-activated protein kinase. J Biol Chem. (2005) 280:39582–93. 10.1074/jbc.M507443200 16176927

[79] Fedele G Di Girolamo M Recine U Palazzo R Urbani F Horenstein AL. CD38 ligation in peripheral blood mononuclear cells of myeloma patients induces release of protumorigenic IL-6 and impaired secretion of IFNgamma cytokines and proliferation. Mediators Inflamm. (2013) 2013:564687. 10.1155/2013/564687 24489445 PMC3892939

[80] Fedele G Sanseverino I D'Agostino K Schiavoni I Locht C Horenstein AL. Unconventional, adenosine-producing suppressor T cells induced by dendritic cells exposed to BPZE1 pertussis vaccine. J Leukoc Biol. (2015) 98:631–9. 10.1189/jlb.3A0315-101R 26089537

[81] Sitkovsky M Lukashev D Deaglio S Dwyer K Robson SC Ohta A. Adenosine A2A receptor antagonists: blockade of adenosinergic effects and T regulatory cells. Br J Pharmacol. (2008) 153(Suppl 1):S457–64. 10.1038/bjp.2008.23 18311159 PMC2268051

[82] Leach DR Krummel MF Allison JP. Enhancement of antitumor immunity by CTLA-4 blockade. Science. (1996) 271:1734–6. 10.1126/science.271.5256.1734 8596936

[83] Nishimura H Honjo T. PD-1: an inhibitory immunoreceptor involved in peripheral tolerance. Trends Immunol. (2001) 22:265–8. 10.1016/S1471-4906(01)01888-9 11323285

[84] Zarek PE Powell JD. Adenosine and anergy. Autoimmunity. (2007) 40:425–32. 10.1080/08916930701464939 17729036

[85] Borcherding N Kolb R Gullicksrud J Vikas P Zhu Y Zhang W. Keeping tumors in check: a mechanistic review of clinical response and resistance to immune checkpoint blockade in cancer. J Mol Biol. (2018) 430:2014–29. 10.1016/j.jmb.2018.05.030 29800567 PMC6071324

[86] Ribas A Wolchok JD. Cancer immunotherapy using checkpoint blockade. Science. (2018) 359:1350–5. 10.1126/science.aar4060 29567705 PMC7391259

[87] Sharpe AH. Introduction to checkpoint inhibitors and cancer immunotherapy. Immunol Rev. (2017) 276:5–8. 10.1111/imr.12531 28258698 PMC5362112

[88] Sharma P Hu-Lieskovan S Wargo JA Ribas A. Primary, adaptive, and acquired resistance to cancer immunotherapy. Cell. (2017) 168:707–23. 10.1016/j.cell.2017.01.017 28187290 PMC5391692

[89] O'Donnell JS Long GV Scolyer RA Teng MW Smyth MJ. Resistance to PD1/PDL1 checkpoint inhibition. Cancer Treat Rev. (2017) 52:71–81. 10.1016/j.ctrv.2016.11.007 27951441

[90] Postow MA Sidlow R Hellmann MD. Immune-related adverse events associated with immune checkpoint blockade. N Engl J Med. (2018) 378:158–68. 10.1056/NEJMra1703481 29320654

[91] Vijayan D Young A Teng MWL Smyth MJ Targeting immunosuppressive adenosine in cancer. Nat Rev Cancer. (2017) 17:709–24. 10.1038/nrc.2017.86 29059149

[92] Chen L Diao L Yang Y Yi X Rodriguez BL Li Y. CD38-mediated immunosuppression as a mechanism of tumor cell escape from PD-1/PD-L1 blockade. Cancer Discov. (2018) 8:1156–75. 10.1158/2159-8290.CD-17-1033 30012853 PMC6205194

[93] Whiteside TL. Targeting adenosine in cancer immunotherapy: a review of recent progress. Expert Rev Anticancer Ther. (2017) 17:527–35. 10.1080/14737140.2017.1316197 28399672 PMC6702668

[94] Mittal D Young A Stannard K Yong M Teng MW Allard B. Antimetastatic effects of blocking PD-1 and the adenosine A2A receptor. Cancer Res. (2014) 74:3652–8. 10.1158/0008-5472.CAN-14-0957 24986517

[95] Arana P Paiva B Cedena MT Puig N Cordon L Vidriales MB. Prognostic value of antigen expression in multiple myeloma: a PETHEMA/GEM study on 1265 patients enrolled in four consecutive clinical trials. Leukemia. (2018) 32:971–8. 10.1038/leu.2017.320 29099494

[96] Moreno L Perez C Zabaleta A Manrique I Alignani D Ajona D The mechanism of action of the anti-CD38 monoclonal antibody isatuximab in multiple myeloma. Clin Cancer Res. (2019). 10.1158/1078-0432.CCR-18-1597. [Epubh ahead of print]. 30692097

[97] Krejcik J van de Donk N. Trogocytosis represents a novel mechanism of action of daratumumab in multiple myeloma. Oncotarget. (2018) 9:33621–2. 10.18632/oncotarget.26098 30263089 PMC6154747

[98] Chillemi A Quarona V Zito A Morandi F Marimpietri D Cuccioloni M Generation and characterization of microvesicles after daratumumab interaction with myeloma cells. Blood. (2015) 126:1849–1849.

[99] van de Donk N Usmani SZ CD38 antibodies in multiple myeloma: mechanisms of action and modes of resistance. Front Immunol. (2018) 9:2134 10.3389/fimmu.2018.02134 30294326 PMC6158369

[100] Young A Mittal D Stagg J Smyth MJ. Targeting cancer-derived adenosine: new therapeutic approaches. Cancer Discov. (2014) 4:879–88. 10.1158/2159-8290.CD-14-0341 25035124

[101] Krejcik J Frerichs KA Nijhof IS van Kessel B van Velzen JF Bloem AC. Monocytes and granulocytes reduce CD38 expression levels on myeloma cells in patients treated with daratumumab. Clin Cancer Res. (2017) 23:7498–511. 10.1158/1078-0432.CCR-17-2027 29025767 PMC5732844

[102] Deaglio S Dianzani U Horenstein AL Fernandez JE van Kooten C Bragardo M. Human CD38 ligand. A 120-KDA protein predominantly expressed on endothelial cells. J Immunol. (1996) 156:727–34. 8543826

[103] Gallay N Anani L Lopez A Colombat P Binet C Domenech J. The role of platelet/endothelial cell adhesion molecule 1 (CD31) and CD38 antigens in marrow microenvironmental retention of acute myelogenous leukemia cells. Cancer Res. (2007) 67:8624–32. 10.1158/0008-5472.CAN-07-0402 17875702

[104] Kumar SK Anderson KC. Immune therapies in multiple myeloma. Clin Cancer Res. (2016) 22:5453–60. 10.1158/1078-0432.CCR-16-0868 28151713

[105] Kumar S Saini RV Mahindroo N. Recent advances in cancer immunology and immunology-based anticancer therapies. Biomed Pharmacother. (2017) 96:1491–500. 10.1016/j.biopha.2017.11.126 29198747

[106] Horenstein AL Chillemi A Quarona V Zito A Mariani V Faini AC. Antibody mimicry, receptors and clinical applications. Hum Antibodies. (2017) 25:75–85. 10.3233/HAB-160305 28035914

[107] Weiner LM Surana R Wang S. Monoclonal antibodies: versatile platforms for cancer immunotherapy. Nat Rev Immunol. (2010) 10:317–27. 10.1038/nri2744 20414205 PMC3508064

[108] Shepard HM Phillips GL D Thanos C Feldmann M. Developments in therapy with monoclonal antibodies and related proteins. Clin Med (Lond). (2017) 17:220–32. 10.7861/clinmedicine.17-3-220 28572223 PMC6297577

[109] Rieth J Subramanian S. Mechanisms of intrinsic tumor resistance to immunotherapy. Int J Mol Sci. (2018) 19:E1340. 10.3390/ijms19051340 29724044 PMC5983580

[110] Ferrero E Lo Buono N Horenstein AL Funaro A Malavasi F. The ADP-ribosyl cyclases– the current evolutionary state of the ARCs. Front Biosci. (2014) 19:986–1002. 10.2741/4262 24896331

[111] Caligaris-Cappio F Bergui L Tesio L Pizzolo G Malavasi F Chilosi M. Identification of malignant plasma cell precursors in the bone marrow of multiple myeloma. J Clin Invest. (1985) 76:1243–51. 10.1172/JCI112080 2931452 PMC424031

[112] Chillemi A Zaccarello G Quarona V Ferracin M Ghimenti C Massaia M. Anti-CD38 antibody therapy: windows of opportunity yielded by the functional characteristics of the target molecule. Mol Med. (2013) 19:99–108. 10.2119/molmed.2013.00009 23615966 PMC3667209

[113] van de Donk NW Janmaat ML Mutis T Lammerts van Bueren JJ Ahmadi T Sasser AK Monoclonal antibodies targeting CD38 in hematological malignancies and beyond. Immunol Rev. (2016) 270:95–112. 10.1111/imr.12389 26864107 PMC4755228

[114] Frerichs KA Nagy NA Lindenbergh PL Bosman P Marin Soto J Broekmans M. CD38-targeting antibodies in multiple myeloma: mechanisms of action and clinical experience. Expert Rev Clin Immunol. (2018) 14:197–206. 10.1080/1744666X.2018.1443809 29465271

[115] Castella B Faini A Yakymiv Y Morandi F Larocca A Oliva S Induction of structural and functional effects of myeloma cells after daratumumab treatment. Cancer Res. 78(13 Suppl):2122 10.1158/1538-7445.AM2018-2122

[116] Franssen LE Mutis T Lokhorst HM van de Donk N. Immunotherapy in myeloma: how far have we come? Ther Adv Hematol. (2019) 10:2040620718822660. 10.1177/2040620718822660 30719268 PMC6348514

[117] Curti A Aluigi M Pandolfi S Ferri E Isidori A Salvestrini V. Acute myeloid leukemia cells constitutively express the immunoregulatory enzyme indoleamine 2,3-dioxygenase. Leukemia. (2007) 21:353–5. 10.1038/sj.leu.2404485 17170728

[118] Mesiano G Zini R Montagner G Bianchi N Manfredini R Chillemi A. Analytic and dynamic secretory profile of patient-derived cytokine-induced killer cells. Mol Med. (2017) 23:235–46. 10.2119/molmed.2017.00084 28805233 PMC5630476

[119] Horenstein AL Chillemi A Zini R Quarona V Bianchi N Manfredini R. Cytokine-induced killer cells express CD39, CD38, CD203a, CD73 ectoenzymes and P1 adenosinergic receptors. Front Pharmacol. (2018) 9:196. 10.3389/fphar.2018.00196 29731713 PMC5920153

[120] Nwangwu CA Weiher H Schmidt-Wolf IGH. Increase of CIK cell efficacy by upregulating cell surface MICA and inhibition of NKG2D ligand shedding in multiple myeloma. Hematol Oncol. (2017) 35:719–25. 10.1002/hon.2326 27430430

[121] Ishikawa T Ito T Endo R Nakagawa K Sawa E Wakamatsu K. Influence of pH on heat-induced aggregation and degradation of therapeutic monoclonal antibodies. Biol Pharm Bull. (2010) 33:1413–7. 10.1248/bpb.33.1413 20686240

[122] Latypov RF Hogan S Lau H Gadgil H Liu D. Elucidation of acid-induced unfolding and aggregation of human immunoglobulin IgG1 and IgG2 Fc. J Biol Chem. (2012) 287:1381–96. 10.1074/jbc.M111.297697 22084250 PMC3256859

[123] Wang T Kumru OS Yi L Wang YJ Zhang J Kim JH. Effect of ionic strength and pH on the physical and chemical stability of a monoclonal antibody antigen-binding fragment. J Pharm Sci. (2013) 102:2520–37. 10.1002/jps.23645 23824562

[124] Wang W Singh S Zeng DL King K Nema S. Antibody structure, instability, and formulation. J Pharm Sci. (2007) 96:1–26. 10.1002/jps.20727 16998873

[125] Dimopoulos MA Oriol A Nahi H San-Miguel J Bahlis NJ Usmani SZ. Daratumumab, lenalidomide, and dexamethasone for multiple myeloma. N Engl J Med. (2016) 375:1319–31. 10.1056/NEJMoa1607751 27705267

[126] Palumbo A Chanan-Khan A Weisel K Nooka AK Masszi T Beksac M. Daratumumab, bortezomib, and dexamethasone for multiple myeloma. N Engl J Med. (2016) 375:754–66. 10.1056/NEJMoa1606038 27557302

[127] Boison D. Adenosine kinase: exploitation for therapeutic gain. Pharmacol Rev. (2013) 65:906–43. 10.1124/pr.112.006361 23592612 PMC3698936

[128] Kawano Y Inada Y Sasano T Nishimura N Hata H Matsuoka M The purine metabolic enzyme AMPD1 is a novel therapeutic target for multiple myeloma. Blood. (2018) 132:5614 10.1182/blood-2018-99-118603

